# 

**Published:** 1888-04-07

**Authors:** 


					(14* X0
The Hospital
A Weekly Institutional Tournal of
Science, flfcebicine, IRuvstng, anb philanthropy.
EDITED BY
HENRY C. BURDETT,
AUTHOR OF " HOSPITALS AND THE STATE" J c< PAY HOSPITALS OF THE WORLD" J t( COTTAGE HOSPITALS, GENERAL, FEVER, AND
CONVALESCENT, WITH FIFTY BEDS AND UNDER I THEIR ORGANISATION, MANAGEMENT, AND WORK " J ETC., ETC.
ACTING EDITOR:
GEORGE W. POTTER, M.D.,
AUTHOR OF PAPERS ON tC BACTERIOLOGY" " SCARLET-FEVER " " RHEUMATISM"; ETC., ETC.
VOL. IV. FOR 1888.
(7TII Atril TO 29TH Septsmber, 1888.)
LONDON:
PUBLISHED BY "THE HOSPITAL" (Limited) 38, OLD JEWRY, E.C.
THE HOSPITAL.
lP\
4J0UA /
INDEX TO VOL. IV. 1888.
Abuse of Hospital Privileges in London, 404
Accidents and Sadden Illness, the Immediate
Treatment of, 352
Aids to Nursings
?   Bathing and Baths, 56, 89
Focd and Feeding, 5
Poultices, Fomentations, etc., 89
Alcohol as a Remedial Agent, 424
Alcohol, Consumption of, at Devon and Exeter
Hospital, 124
Hull Royal Infirmary,
29
Lendon Temperance
Hospital, 164
Alcohol, Professor Biny on, 213
Alcohol versus Race, 250
Ambulances Wanted 343, 359, 388
American Jo tings, 72, 118
American Physicians and Surgeons' Congress, 45
Amusements with Profit for All Ages, 16, 36, 52,
68, 84, 100, 116, 132, 172, 1S8, 204, 220, 236,
252 268, 282 300, 332, 348, 364, 380, 412, 428
An Automatic Quack, 67
Animals' Institute, an, 228
"An Ishmaelite," by Leslie Keith, 13, 33, 49, 65,
8 r, 98, 113, 128,168,185,201, 217,233,249
265, 282, 297, 313, 329, 345, 361, 393, 377, 409,
425
Anonymous Slanderers and Working Men, 189
Anti-Vaccination Tactics, 279
An Unvarnished Tale, 382
Army Recollections, 296, 306
Art, a New, 194
Asylum Attendants, 127
Asylum Romince, an, 411
Attempt to Blow up a Small pox Hospital, 76
A Word to M.P.'s, 237
Barntt, Cottage Hospital for, 24
Bath, Best Time for Taking a, 99
Bath for the People's Palace, 109
Baths and Washhouses at Hampstead. 372
Bethlem Royal Hospital for Lunatics, Medical
Staff of, 389
Blackburn Infirmary, 405
Blackheath Institute for Trained Nurses, 341
Bl.yth, Mr. J. S., Death of, 77
Bolingbroke House, 192
Brag or Benevolence ? 423
British Medical Association, 317, 325, 327
Briiish Nurses' Association, 15, 256
Bones and Backbones, 84
Bristol Children's Hospital, j6
BrUtol Infirmary, 324
Bristol Royal Infirmary, Hon, Staff Appoint-
ments, 29
British Home for Incurables, 91
Burdett, Mr. H. C., on the Pay System at
Hospitals, 400
Buried Alive, 69
Buxton Bath Ctiarity, 109
Cancer. Popular Fallacies about, 85
Cancer Hospital, a Garden Party at the, 394
Cancer Hospital, Brompton, 93,375
Cancer, the Bacillus of, 264
Celebrity, Danger of, 50
Celt and the Teuton, the, 215
Centenary of York Dispensary. 24, 31
Certain Knaves and Certain Fools. 215
Charitable Relief in London, 261
Charity Organization Society, 397
Cheap Houses for Ladies, in
Cheltenham General Ho-pital, .02
Children, Employment or, TJnd;r Fourteen, 357
Ch'ldren and Cri.ne, 366
Children's Country Hospitals, 171
Child Cheats, 311
Children. Insurance of, 311
Christianity and the Sick, 151
Cholera Precautions, 321
Churchill, Lord R., and St. Mary's Hospital, 92,
103, in
on Our Hospital System, 101,
103
City of London Asylum, 89
Climate and Suicide, 344
Clothing, Sanitary Woollen, 42
Committees, Good-natured, 347
Committeemen and Business Contracts, 423
Congress of American Physicians and Surgeons at
Washington. 45
Constitution Building, 39, 55 7r, 122
Convalescent Home at Sydney, N.S.W., 29
Consumptives, the Care of, 183
Consumption Hospital, Brompton, 323
Constructive Charity, 397
Cottage Hospital for Barnet 24
Crown Prince William and His Left Arm, 259
Daily Telegraph,-, and Our Bachelors, 199
Daneries, the, 91
Diet Scale, Suggestions for, 32
Diets, the Battle of, 407
Difficult Cases, i s
Dispensers' Column, 175, 202
Doctor's Pulpit, the :?
A Fresh Start with the
Summer, 160
A Day in Bed, 193
\ Question of Smoke, 207
Constitution Building, 39,
55, 71, 122
Heat Stroke, 319
Human Barometers, 287
In the Midst of Life, 305
Marriage and Moral?, 369
Marriage?or What ? 353
Mental Depression, 415
Middle Age, 399
Nerve Storms, 3
Nerve Tides, 21
The Choice of a Hea'th
Resort, 223
The Uses of a Health Resort,
238
Tonics, 175
Voluntary Imbecility, 339
Waste of brain Resources,
27 i
Doctors and Chemists, 391
Donegal Fund, 183
Dorset County Hospital, Misunderstandings at,
93, 127, 263
Dundee Intirmary, Death of a Girl at, 9
Edinburgh Charities, Legacies to, 324
F.ditor's Letter-box, 58, 99, 418
Elberfeld System, the, 53
Ellis, Rev. F. W., Death of, 29
Embalming, the Art of, 366
Emperor, the Dead, 255
Everybody's Page, 10, 30, 46, 62, 78, 94. no, 126,
166, 246, 262, 278, 294, 310, 326, 342, 391, 374,
406
Explanations and Suggestions, 15
Facts and Figures, 152
False Issues and Fanciful Facts, 337
Family Feeling, the Decay of, 199
Faults on Both Sides, 127
Fees, 379
Fever Precautions, 321
Firs Home, Bournemouth, 61
Flat Feet, 264
Folkestone, Proposed Site of New Hospital at, 341
Free Rest for Hospital Nurses, 123
Friends' Retreat at York, 48
Friendly Societies, Profane Demonstrations of,
247
French Hospital, th^ New, 308
Fresh Fruit as Food, 407
German Hospital, 41
Germany, Floods in?Mansion House Fund, 260
Girls' Friendly Society, 33
Gladstone, Mrs., on our Hospitals, 159
Glasgow Rojal Infirmary, Painful Incident at, 108
Glasgow. New Victoria InSrmary, 245
Glasgow University Presented with a Club House,
Graham, Mr., Bequest to London Charities, 67
Grantham Hospital, 63
Gravesend Hospital and District Nurses, 180
Great Northern Central Hosp tal, 41, 92, 124, 253,
261
Governess, Protest of a, 99
Hanging, the Case for, 183
Hard Case, a, 58. 99
Healihy Hospitals, 83
Help, a Case lor, 75
Hereford County Asylum, 48
Hints for the Sick Room, 224, 240, 257, 291, 360,.
_392> 370..4?3 4_'6
ilidai
Holidays and the Weather, 343
Home Colonization, 57
Homoeopathic Earl, an, 63
Homoeopathy, the Odium Medicuai and, in
America. 306
Honorary Staff Appointments at Bristol Royal
Infirmary, 29
Horse Ambulance for Leeds Royal Infirmary, 77
Hospital Clothing and Bedding, 396
Hospital Extravagance, 388
Hospital Finance, a Disclaimer, 350
Hospital for Sick Children, 76
Hospital Life, Bright Side of, 99
Hospital Patients and Stimulants, 63
Hospital Opinion, the Growth of, 173
Hospital Saturday in Birmingham, 124, 228, 229
Colchester, 309
Hastings, 341
Metropolitan, 197, 260, 261, 276,
356, 372
Movement, 54, 76
Our First, 232
Hospital Sunday, Awards and Criticisms, 275
Metropolitan, 134, 173, 181, 286
Reading, a Homily for, 157
Preachers and Subjects for, 158,
162
Hospital System, a Defence of the Old, 303
The Needs and Backbone of our,
102, 103
Hospital Workers, a Census of, 16 2
Hospitals Association, Annual Meeting, 124
Life Members, 108,164,196
228
Mr. L. B. Willoughby
Appointed Secretary, 108
Session 1888-9, 3^8
Session 1887-8, Papers read
?Spi cial Meeting, 180
at, 44 I
The Pay System at Hospi-
tals, 79, 8', 96, 97, 98,400.
THE HOSPITAL. iii
Hospitals, a Few Words f^r Our, 159
Hospitals and Medical Charities of London, a
Year's Work in, 153
Hospitals and the Drama, 188
Hospitals, Comparative Expenditure of, 32 51
Hospitals, M itropolitan, Stated Mismanagement
at. 189
Cost per Occupied Bed
in, 335, 349
Hospitals on Provident Principles, 167
Hospitals, the Claims of, 351, 367
Hospitals, the General Practitioner's Indictment of,
221, 269
Housekeeping, th? Science and Art of, 304
How to be Healthy, 9 c
How to Grow Beautiful, 334
How to Live Long, 389
Hull Borough Asylum, 88
Hull Royal Infirmary, Consumption of Alcohol at,
? 29
Hydrophobia and PaUeurism, 79
In and Out Among the Hospitals :?
Bradford Infirmary, 371
? Cheltenham General Hospital, 102
City of London Hospital, 208
General Hospital, Birmingham, 307
Glasgow Royal Infirmary, 402
Halifax Infirmary, 402
?  Italian Hospital, 163
Kilmarnock Infirmary, 43
? King's College Hospital, 208
Leicester Infirmary, 402
London Hospital, 102
Manchester Royal Infirmary, 371
Middlesex Hospital, 163
Newcastle-on-Tyne Royal Infirmary,
371
Norfolk and Norwich Infirmary, 43
Paisley Infirmary 402
Pennyslvania Hospital, 163
Queen's Hospital, Birmingham, 248
Radcliffe Infirmary, Oxford, 43
Royal London Ophthalmic Hos-
pital, 307
? St. George's Hospital, 248
Splendid Special Hospital, 307
Suffolk General Hospital. 208, 248
Westminster Hospital, 248
West London Hospital, 307
India, Female Medical Aid to the Women of, 477
Infectious Cases, Temporary or Permanent Hos-
pitals for? 11
Infectious Hospitals, Report of Statistical Commit-
tee, 293
Inquisition, a New, 350
Insane, Mechanical Kestraint of the, 405
International Medical Congress, 308
Invalid Children, Home for, 92
Ireland, Cottage Industries in, 295
Irish Exhibition, 167
Jaeger Revolution, the 42
Jubilee Cottage Hospital at Barnet, 24
Ki'marnock Infirmary, 43
King of Causes of Disease, the, 373
King's College Hospital, 76
Knowledge, the Obligations of, 17
Labourers' Wages and Labourers' Homes, 8?, 105,
"9
Lady Dufferin's Fund, 4
Lady Visitors at Hospital, 277
Leeds Royal Infirmary, Workmen's Jubilee Gift, 77
Leeds Infirmary, Extension of, 279
Legacies to London Hospitals, 404
Legislative Social Reforms, 117
Leicester Infirmary, Memorial to Mr. George
_ Pearce, 373
Leicester Borough Asylum, 48
Leicester Dispensary, Charges against the Manage-
ment of 9 107
Leicester Provident Dispensary, 171
London Charities, Quinn's and Graham's
Bequests, 67
London Hospital, Facts about the, 23
Quinquennial Appeal, 45 102
A Plea for the, 59. 174
Patients' Bill of Fare at, 277
? Public Meeting in Aid of, 229
London Hospitals, and Metropolitan Members of
Parliament, 1
London Skin Hospital, First Report of, 244
Long Life, an Apostle of, 263
Lozier, Dr. Clemens, Death of, 75
Lucifer, a Temple of, 12
Lunatic Asylums for Middle Classes, 341
Lying-in Hospitals?Home for Women Discharged
from, 324
Lying-in Hospitals, Medical Press and Circular,
and Mr. Ryan's paper on, 47
Lying-in Hospital, the City of London, 164
Madness and Great Wit, 222
Madness or Vanity, 398
Maimed by Nature, 51
Malingering, 289, 315, 39s
Many Ways of Resting, 231
Manchester, Small pox at, 293, 308
Manchester, Overcrowding in, 388
Manchester Infirmary, 37}
Massacre of Innocents, 263
Matches, the Manufacture of, 12
Medi al Knowledge, the Value of a Little, 343
Medical Olive Branch, a, 317
Medical Plan of Campiign, 333
Medical Profession and Income Tax, the, 299
Medical Schools and Medical Students, 383
the Discipline of, 385
Medical Prodigy at Glasgow, a, 31
Medical Study, Minor Aidsto, 194
Met'opolitan Provident Association, 293
Medicine Men of the World, 184
Midwives, are they Despised ? 31
Middlesex Hosp'ual and Out Patient Department,
61
Festival Dinner, 109
Miller Hospital, Greenwich. 260
Misunderstandings at Hospitals, 93, 263
Memory, Aids to, 302
Moderate Drinking. 9
Money Value of a Live Man, 359
Moxon, the late Dr., Memorial to, 357
Murder by a Child, 372
Muidered Children, 391
National Pension Fund for Nurses, Appointment
of Manager, 292
and its one Adverse
Critic, 37s
National Pension Fund for Nurses, 181, 302, 383
401
National Hospital for Paralysed, etc., 196, 244
Nerve Storms, 3
Nerve Tides. 21
Newcastle Infirmary, 45
Newcastle Royal Infirmary, a Ladies' Committee
for, 228
New Remedies and Appbances. 259, 299, 418
North Londo.i Hospitalf"r Children, 61
North Wales Asylum, Denbigh, 48
Norfolk and Norwich Infirmary, 43
Norfolk and Norwich Hospital, 277
North-West London Hospital, 261
Nottingham Borough Asylum, 23
Nottingham County Asylum, 89
Curses, a Plea for the, 15
Nurse-Training Schools and Registration, 37, 285
Nurses in Rebellion, 109
Nursing of Bachelors, the, 167
Nursing Home at Cairo, 15
Nurse Farming, 247
Open-door System, 7
Opiates and Anaesthetics, 238
Out-patients and Annual Subscriptions, 171
Owens Cottage, Storm at, 327
Oxford County Asylum, 88
Parasitic Diseases of Skin, Galvanic Treatment,
379
Paralysis after Diphtheria, 264
Parliament, Metropolitan Members of, and Lon-
don Hospitals, 1, 237
Pasteurism and Hydrophobia, 79
Patient, the Plea of the, 387
Patients' Contributions to Hospitals, by Mr. W.
Burdett-Coutts, M. P., 81, 96,97, 98, 113. 127,
131, 161, 176, 191, 205, 209, 219, 225, 229J 241
Paupers for Farmers, 295
Pay System at Hospitals, 79, 81, 96, 97, 98, 112,
127, I3t, 161, 176, 191, 205, 2?9i 2I9> 225> 229>
241, 400
Paying Patients and General Practitioners, 219
People's Palace, 109, 309
Phlebotomy in Excelsis, 242
Phrenology. 234
Physicians, Presidency of the College of, 11
Physiology and its Uses, 22, 74, 195, 280, 312, 417
Physical Improvement and Technical Training,
120
Pity the Poor Practitioner, 311
Play, 169
Poetry :
A Hypochondriacal Patient's Song. 396
An Indian Woman's Tribute to Lady
DufTerin, 331
Four Worlds, 2
Frederick III., Emperor and King, 203
Land of Golden Day, 43
Lines to a Deserted Study, 51
Lonely, 299
Operations, 378
Out of the Depths 20
The Old and the New, 190
The Puddingless Patients, 258
To E. P. M., 14
'. enus or jEscalapius, 206
Poor John Aldcroft, 359
Poor R lief and Tolal Abstinence, 47
Portents and Epidemics, 414
Presidency of the College of Physicians, 11
Proctor. Death of Mr. Richard A., 405
Provident Dispen-aries, 8
-?  Dispensary System at Sunderland, 9
Special Hospitals, 215
and Improvident Hospitals, 253
Queen's Jubilee Offering. 60
Quinn, Mr. Henry, Bequest to London Charit es
67
Radc'iffe Infirmary, Oxford, 43
Registration of Nurses, 170, 271
Registration and Nurse-Training Schools, 37
Research Laboratory, /J, 410
Reviews : " Margaret Dunmore," 35
Laryngoscopy and Rhinoscopy," 35
" Our Hundred Days in Europe." 64
" Human Anatomy and Physiology," 65
" Ismay's Children," 129
" Medical Nursing," 130
?' Prosperity or Pauperism," 130
" New Judgment of Paris," 216
" Posological and Therapeutic Tables,"
216
" Amputations Treated Antiseptically
267
" Science and Art of Surgery," '.67
?' A More Excellent Way," 281
" Specific Febrile Disorders, Abortive
Treatment of," 281
" In Hot Haste," 3^5
" Chemistry, Students' Handbook of,"
355
" Dying Scientifically," 33/
' Out of Work.' 321
" Fancy Fair Religion," 32-
" The Intestinal Diseases of Childhood,"
424 ^
" Life in the Cut, 424
Richmond Hospital. 93, 244
Romance, An Asylunri, 411
Rosebery, the Earl, on Voluntary versus State-
Supported Hospitals, iq
Roses in the Rain, 279
Rotherham Hospital, Calmer Weather at, 95
Round About the Asylum? :?
Abergavenny Joint Counties Asylum,
179
Aberdeen Royal Asylum, 290
Bucks County Asylun, 179
Carmarthen County Asylum, 200
Ciiy of London Asylum, 89
Friends' Retreat, York, 48
Gloucester, Barnwood House, 179
Gloucester County Asylum, ?go
Hereford County Asylum, 48
Hull Borough Asylum, 88
Kiliarney District Asylum, 200
Lancashire Asylum, Lancaster Moor,
336
Lancashire Asylum, Pres'.wich, 336
? Lancaster County Asylum, Rainhi
290
Leicester Borough Asylum, 48
Lincoln County Asylum, 200
Middlesex County Asylum, Ban-
stead, 336
Middlesex County Asylum, Han-
well, 323
Newcastle-upon-Tyne Asylum, 290
Northampton County Asylum. 227
North Wales Asylum at Denbigh, 48
Nottingham Borough Asylum, 23
???   Nottingham County Asylum, 88
Oxford County Asylum, 88
Private and Pauper Asylums, 227
Stafford County Asylum, 200
? Stafford County Asylum, Lichfield
290
Sussex County Asylum, 227
Warneford House, Oxford, 323
Wilts County Asylum, Devizes, 323
Worcester County Asylum, 323
Royal Free Hospital, Death of the Secretary, Mr.
? Blyth, 77
Appointment of Secretary,
196
Royalty and Hospitals, 196, 260
Saccharin for Diabetics, 379
Sailors and Anti-Scoi buues, 107
St. Catherine's Hospital, 235
t. Mary's Hospital, W., Death of the Chaplain
29
St Mary's Hospital, the Site of, 1:1
Festival Dinner, 92, 101,
103, XII
Dramatic Performance in airf
o", 276
and Ambulances, 38)
iv ' THE HOSPITAL.
St. Thomas's Hospital, 76 405
Scarlet Fever on Board Training Ships, 24
Scotch Physicians and Surgeons, 6, 40, 73, 186
Scottish Hospital, a Great, 426
Seamen's Hospital Greenwich, Concert to the
Patients, 29
Seaside Season, 318
Self-help Among Nurses. 301
Sewage Question in London, hi
Shaftesbury House, 109
"Silver Fete" in Aid of Victoria Hospital for
Children, 45
Small-pox Hospital, Attempt to Blow Up a, 76
Sources of, 95
Statistics, Swiss, 346
Something Like a Secretary, 63
Spa, an Old and New English, 211
Speakers and Speech, 133
Specialists Again, 407
Squinting and Worms, 379
Stable Door, the, 47s
State-Supported versus Voluntary Hospitals, 19
Stimulants and Hospital Patients, 63
Student's Column, 65, 115, 188, 274, 316, 328,
347, 363
Student's Bitter Cry, the, 383
Sturge, Mr. Geo., the Will of the Late, 197
Stretchers, Proper Mode of Carrying, etc., 276
Sussex County Hospital, 93
Sunderland Infirmary, 108
Summer Underclothing, 231
Australia, Notes from, xci
British Nurses' Association, lxxv
Chastisement, Well-deserved, xix
District Matron's Diary, a, xi, xliii, Ixiii
District Nursing, lviii
Everybody's Opinion :?
British Nurses'Association, vii
Censoriousness, lxxxiii
Courage, the Question of, xcix, cii
District Nursing, lxxv
Midwifery, Free Training in, vii,
lxxxviii
Nice, the Hollond Institution, vii
Nuise, the Life ot a Private, lxxi
Nurse, the Title of, lxxxviii
Nurses, Long Hours, etc., xxvii
Nurse's Position, a, cii
Nurses, the Pay of Private, xlviii, lv
Nurses and Patients, Ii, lv
Nurses for Mental Disorders, lxiv
? Nurses, George Eliot's Thoughts on,
Ixvii
Nursing and Emotion, xcix
Surgery of the Past, 254
Suicides by Carbolic Acid in Liverpool, 404
Surgical Operation, a New, 322
" Sweated " Lady, a, 423
Sweating System and Nursing Sisterhoods, 124
Sydney, N. S. W., Convalescent Home for, 29
Technical Training and Physical Improvement, 120
Thunderstorms and Lightning Accidents, 235
Total Abstinence and Poor Relief, 47,
Tuberculosis, Swedish Commission to Investigate
Nature of, 333
Tuberculosis, 379
Unemployed, The Paddington Fund for, 251
Universal Review and the Lancet, 203, 270
University College Hospital, 9
University for Lcndon, 95
Unsavoury Subject, an, 111
Vanity or Madness, 398
Vegetarianism, 5T
Victoria Hospital for Children, Silver Fete, 45
Victoria Park Chest Hospital, Unauthorised Dis-
section at, 125
Vinnicombe Bequest, the, 135
Voluntary Chaplains, 413
Voluntary versus State-Supported Hospitals, 19
Voyages in Vacation, 419
Walsall Cottage Hospital, Conduct of the Sistirs
at, 8
" Watchman, What of the Night ? " 91
Wealth for Women, 23T
Western Ophthalmic Hospital, 293
West-end Mews, The Doctor in the, 408
Westminster Abbey, Coronation Commemoration
Service, 197
Whitechapel Murders and the Rich, 391
Will Workmen Give? 79
Within the Wards, 264, 288, 379
Wolverhampton General Hospital, 164
Women and Faddists, 47
Women, Illused, 247
Women, London School of Medicine for, 3-8
Women, Mr. Walter Besant's Craze about, it, 47
Women's Thrift, Lord Wolseley on, 199
Women's Jubilee Fund and Nursing the Sick Poor,
228. 309, 327
Workhouse Infirmary Nursing Association, 165
Workmen, Progress of Self-heip Among, 295
Workmen's Contributions to Hospitals, 79, 332
Words of Consolation : ?
? The Sufferings ot Christ, 2,
18, 33, 70, 86
Vork Dispensary, Centenary, 24, 31
THE NURSING MIRROR.
Everybody's Opinion:?
Nursing at Reduced Rates, Ixxxviii
Nursing for the Middle Classes, lxiii
Nursing Ovariotomy Cases, cii
Nursing, Private, lxxix
Old Nurses and New, lxxv
?  Patients and Visitors, lx
Presence of M ind versus Panic, xliv, li
Probationer's Protest, a, xv, xx,
xxiv, xxxviii, lvi
Red Cross, the, xcvi
Vocation, a Mistaken, lxxi
Volunteer Nurses, ciii
Weak Anliles, the Cause of, lxxxiii
Workhouse Nurses, xxxviii
Zenana Medical College, the, lxxxi,
xcii, xcvi
Glasgow Royal Infirmary, xcv
Gratitude, a Touching Story of, lxxxiii
Ipswich Nurses' Home, Ixii
National Pension Fund for Nurses, i, vi, x, xiv,
xviii, xxii, xxxiv, liv
Notes from America, xlii, xlv i, liv
Nurse, the Life of a Private, lxii
Nurses' Bookshelf, the, xlvii, lii, lxiv, lxvii, Ixxx
Nurses' Home. Ipswich, lxii
Nurse's Note Book, a, xxvii
Original Articles:?
a Nurses' Provident Fund, xlvii
??   a Nurse's Progress, lxxiv, Ixxviii, Ixxxii
a Sister's Holiday, xciv, xcviii
Country Nurses, xxvi
Nursing the Insane, xxxix
Nursing in New York 1
Nurses in Novels, lxvi
Old and New Nurses, xlii
Peculiar Patients, lxxxvi
Sick Nursing Among the Poor, xc
The Crimea, lxxxvi
The Progress of N ursing in New York Ixx
Philadelphia Directory for Nurses, vii
Probationer, Letters from a, Ixxxvii, xci, xcv, c'i
Registration of Trained Nurses, xxvi
Saint Marylebone Infirmary, Notting Hill, lix
Sick Room, Hints for the, lxvi
spi.
400
The art of not hearing should be learnt by all. It is fully
as important as a cultivated ear, to obtain which both money
and time are spent. There are so many things which it is
painful to hear, so very many which, if heard, will disturb
the temper, that it would be well if we could all be educated
to take in or shut out sounds according to our pleasure.
THE HOSPITAL. April 7, i{
SPECIAL NOTICE TO NEWSAGENTS, ADVER-
TISERS, AND THE TRADE.
OIIAN3E OF OFFICE AND PUBLISHER.
Notice is hereby grven that the Office of The Hospital will hence-
forth be at 38, Old Jewry (Cheap ide end), E.C., to which address all com-
munications should be sent. Mr A. Doig has been appointed Advertising
Agent, and Mr. J. H. S. Hanning, Manager. All Cheques and Post Office
Orders should be made payable to J. H. S. Hanning. 38, Old Jewry, E.C.
crossed Lloyds, Barnetts, and Bosanquets Bank (Limited).
TO SUPERINTENDENTS, SECRETARIES, AND
MATRONS.
The Editor will be glad to have a copy of the Regulations, and also of
every Report issued from the Press of Asylums, Hospitals, Conva'escent and
Nursing Institutions, as well as those of other Charities, so soon as they are
published, a- d an early copy in duplicate of all appeals for funds. Notices
of Annua' and other Meetings, Festival Dinners, etc.. will be welcomed.
All such communications should-be addressed to The Editor, The Lodge,
Porchester Square, London, IV. Any superintendent secretary, matron,
or other chief official who will regularly send such documents and
information will be registered to receive free of cost a cover for binding The
Hospital in half-yearly volumes on sending name and address to the
publisher. ?
A HINT TO THE PUBLISHERS.
A Constant Reader writes : " Would it not be helpful in your
review of books in The Hospital to mention the price ? As
a case in point, I should be glad to know the price of Dr.
Basil's ' Care of Common Diseases,' and one might be glad to
order a book were the price known without the trouble of
writing to the publisher. Also ' My Heart with Thy Heart.'"
We have tried to secure the prices of the books, but have not
succeeded. Perhaps the publishers will now take the hint by
mentioning the price when they forward a book for review.
DIFFICULT CASES.
[Replies endorsed "Difficult Cases," should be addressed to the Editor.]
In answer to " Difficult Cases " I strongly recommend the
poor young woman who has lost the use of her arms to try
for admission into St. Peter's Home, Kilburn, N.W., where
she will receive every possible comfort, and be able to
remain any length of time if incurable. Write for rules to
the Mother Superior, St. Peter's Home, Kilburn, N. W.
Jane Wenlock.
St. Jolin's House, Marine Parade, Lowestoft.
March 29tli, 1888.
Can anyone tell me of a home or institution where a young
woman (aged 28) would be taken who, through chronic rheu-
matism, is unable to earn her living ? The young woman
could help a little in housework, and also do plain needle-
work. She has no relations but a younger brother and sister,
and unless some home can be found for her must go into the
workhouse. E. J. Garrett.
.12, Highbury Crescent.
QUESTIONS AND ANSWERS.
SILVER EAR DRUMS.
The Silver Ear Drums mentioned by Sister Grace may be
obtained from Dr. Nicholson, 15, Camden Park Road, who is
the maker (patent). Nurse Grace.
16 THE HOSPITAL April 7, 1888.
Amusements with Profit for all Ages.
Rules.?All answers must be addressed to the Prize Editor, The Hospital, 38, Old Jewry, Cheapside, London, E.C., not later than
t\e first post on Thursday next. The full name and address must in all cases be given, but a nom de plume may be added for publication. Only one side
of the paper must be written on.
FOR MEN AND WOMEN.
Itulc.?Competitors can enter for all quarterly competitions, but no com-
petitor may take more than two quarttrly prizes during the year.
Acrostic Competition.
Second quarter commenced February 25th, 1888; ends May 19th, 1888.
A PRIZE of ONE GUINEA will be given to the competitor who gains
the highest number of marks for best original^ acrostics during the ensuing
quarter. All acrostics sent in for this competition will be credited accord-
ing to merit, twelve marks being the highest score given for one acrostic.
A PRIZE of HALF-A-GUINEA to be given to the competitor who gains
the highest score for solution of acrostics set. One mark only will be
given to the competitors who solve all the lights of each acrostic.
Exception will be made in the case of quotation acrostics, when two marks
will be given, one for the correct solution of all lights, and one mark
for the source of the quotations.
This week credit will be given for
No. 4.?Double Acrostic.
My second is my first, and vivisectors
Protect themselves with it from all objectors.
x. In ancient time,
In physic's prime,
When alchemists had notions,
With air sublime
And gingling rhyme
They drew from me their potions.
2. You here no ball-room's vain display survey,
The dusky figures wave and sway, then stay ;
Their arms they bend in natural play this way.
Their feet beat time, so peaceful they and gay.
3. Gaze upward to the azure sky,
Where fleecy clouolets scud and fly ;
Here is the kingdom owned by me,
Unrivalled in inr.mensity.
4. In Carthage old, trained not to love
The daughter of brave Hasdrubal,
Her lover's death was said to move
To die of love beneath its wall.
5. With stealthy tread and lowered head
My prey I stalk,
And none may baulk
M y dash and crash like lightning's flash.
6. A Christian convert, Afric's glory,
Here lived and wrote his wondrous story.
7. I come as sinks the sun,
I dance when day is done.
I haunt the enchanted ring,
And voiceless rhythyms sing.
8. Shade of the noble past,
They shattered limbs too fast,
Have mouldered in decay ;
And yet thy beauties last
For ever and a Hay.
9. A thief profane ! yet monarchs share the rpoil,
And draw rectorial tithes from sacred soil.
10. A schoolboy's vice, and yet a master's task,
But teacher's say 'tis ignorance's mask.
Answers.
No. 3 Double Acrostic.
Proem. " In Memoriam." Tennyson.
E ndo W "Pope."
M achiavell I "Byron.'
P hilome L "Drayton."
E ba L " Keble?Christian Year."
R ienz I " Byron?Chtlde Harold."
O pheli A "Shakespear?Hamlet."
R efor M "Carlyle."
Correct answers have been received from Reldas (1), Rowdy Corner (1),
Mater (1), Gretta (1), Lady Clara (1).
(A) THE WORD COMPETITION.
Second Quarterly Competition commenced
February 4th, 1888; ends April 28th, 1888.
Three prizes of one guinea, 15s. and 105. 6d. will be given each quarter to
the three competitors who obtain the highest number of marks; (1) by
making the largest number of words derived from the word or phrase set
for anatomical dissection j or (2) for the best answers to (BJ ; or (3] by (1)
and (2) combined.
We shall give the newspapers and periodicals for dissection during this
quarter, that for this, the ninth week of the quarter, being
" Blackwood."
Observe.?Proper names, abbreviations, foreign words, words of less than
four letters, and repetitions are barred; plurals, and past and present par-
ticiples, of verbs are allowed. Nuttall's Standard dictionary only to be
used.
N.B.?All words sent in must be arranged alphabetically, with correct
total affixed.
Results ot Eighth Week.
Church Times.? Patience (282), Grttta '241), Sym (236), Ellice(228),
Lodge (225), Lightowlers (223), Dcllie (218). .Lauriston (217), E.E. (208),
Duncei2o6), Nelle (186), Brisbane (183),Butterfly (150), Oliver (106).
(B) THE LITERARY COMPETITION.
Some people hate puzzles of all kinds, including word competitions, but
they are fond of correspondence, and many sisters and mothers are
continually receiving and sending letters to and from members of the
family who are abroad in India or the Colonies. The constant coming and
going between England and other portions of the British Empire make it
a. matter of interest and importance to know something about the life, the
climate, the amusements/the adventures, the clothing, and the surroundings
of those who seek a livelihood beyond the seas. We shall therefore try to
find space for the publication of selected letters from abroad, which may
bs sent by any of our readers who have friends and relations in foreign
lands. We shall give marks for these letters as well as for the word compe-
titions which will count equally for these prizes, so that anyone may enter
for both or either, the prizes to be given to those who get the highest
number of marks.
letters from Across the Sen.
Lightowlers sends the following extract from a letter from Grahamstown,
South Africa:?
We do not work among the natives now, but hope to do so when we have
more helpers ; the white people are enough for us; all the blacks we see are
the servants, and they are not very numerous in the town, living more out
on the farms where all the labour is done by Kaffirs, the white farmer over-
looking them The Kaffir race is a very low one ; they are very ugly, with
thick lips and quite black ; the Hottentots are much better looking, and so
are the half castes who form the female servant class. We have no coloured
children in our school or home, as they cannot be mixed with the whites.
Our orphans are the children of Colonists who have deserted them, or have
been killed. It is a common practice here of men to marry, and then leave
their wives to look for woik at the Diamond Fields or at the sea.port towns,
and never return. Then the wife has to work for herself, the children starve
or go to utter ruin. In some cases the parents have died ; then we adopt
the little ones It is a good work, and will prosper I feel sure. Next year
we hope to collect enough money to build on to the house and keep more,
but we want a large sum to do that, and it takes time to gather.
THE CHILDREN'S CORNER,
PRIZES.
FOR MOST CORRECT ANSWERS TO PUZZLES.
This Commenced October ist, 1887.
One Pound a Month.
A PRIZE OF THREE GUINEAS
to the Competitor who shall have sent in the largest number of correct or
best answers during the twelve months.
Puzzles?First Week.
No. 1. Buried Birds.?1. On this wall owls perch at night. 2. Mary
went off in cheerful spirits. 3. Let them use lard for butter. 4. Children,
how late you are.
No. 2 Numerical Puzzle.?I consist of four letters, multiply my first
by five and you will find my last ; halve my first, and place the half as my
third ; my second is a figure of itself worthless.
No. 3. Double Acrostic.?
1. Part of a coat and the reverse of caress,
2. A favourite dog which all sportsmen possess,
3. A canton most fair where mountains abound,
4. A soldier's dettnce when foemen surround,
5. A grand modern show has this classic name,
6. A French town from which comes the choicest champagne.
No. 4. Charade. My first is a respiration, my sccond a place well-
known in ancient history, my whole a pain worse than toothache.
Results oi Fourth Week Puzzles.
No. 13. Arithmetical Puzzle. Fach had ?125 a-year, John spends
^,100 and saves ,?25 ; Charles spends .?180 and gets ,?55 in debt .". 55 + 4=
22^, the amount Charles finds himself in debt at the end of four years.
No. 14. Double Acrostic.
M alio W
A rgal I
R owa N
C hickwee D
H ippopotamu S
No. 15. Enigma, A name.
No. 16. Diamond Puzzle.
B
KEN
FIELD
B E N T L E Y
BEE THOVEN
OCTOPUS
DAVIS
LEE
N
All right.?A. C. K., Sqeaker, Bookworm. Three right?Jumps,
Scrooge, Dame Durden, Bagshot. Two right ?Plummy Fluff, Gem,
Spider, Excelsior, Mount Edgecumbe. One right.?Babette.
Conditions.
I. Every paper sent in must be clearly written, and one side only must
be used. All competitors must mark at the head of their papers the number
of their competition.
II. Each paper must be signed by the author with his or her real name
and address. A nom de plume may be added, if the writer does not desire
to be referred to by us by his real name. In the case of all prize-winners,
however, the real name and address will be published.
III. All communications relating to prize competitions should be addressed
to "The Prize Editor, The Hospital, 38, Old Jewry, London, E.C."
Competitors are requested not to include in letters, etc., to the Prize Editor
communications on matters of other business. All letters must be fully
prepaid.
IV. The Prize Editor of "The Hospital" is the sole judge of the
competitions, and his decision is in all casts final and without appeal
V. The Prize Editor restrves the right to publish any paper sent in,
whether it receives a prize or not. He does not hold himself responsible
for any MSS. sent, nor can he undertake to return rejected competitions.
VI.?All children resident at school are requested to forward their home
address in addition to the school one, when entering for the " Children's
Corner " Competitions.
Notice to all Correspondents.?N.B.?All letters referring to this page, which do not arrive at 38, Old Jewry, Cheapside, London, E.C., by
h z first post on Thursdays, and are not addressed PRIZE EDITOR, will in future be disqualified and disregarded.
No one will be permitted to win the Monthly Prizes twice in any one year, the Annual prizes being offered especially to prevent this.
THE NATIONAL PENSION FUND FOR NURSES.
Incoiporated February 17th, 1888. Established October 16tb, 1887-
PATRONESSES.
The Countess of Rosebery. | Lady Rothschild.
VICE-PRESIDENTS.
The Earl of Aberdeen. I Sir Edmund Hay Currie. I E. A. Hambro, E^q.
Lord Rothschild. | Henry Hucks Gibbs, Esq. | Junius S. Morgan, Esq.
THE COUNCIL.
Walter H. Burns, Esq., Chairman.
Henry C. Burdett, Esq. (The Founder), Deputy Chairman.
J. S. Bristowe, Esq., M.D., F.R.S.
Thomas Bryant, Esq, F.R.C.S.
R. Brudenell Carter, Esq., F.R.C.S.
F. C. Carr-Gomm, E.q.
Percival A. Nairn, Esq.
Edward Rawlings, Esq.
Major Ross, M.P.
Alfred Charles de Rothschild, Esq.
J. C. Steele, Esq., M.D,
John Wainey, Esq.
Clifford Wigram, Esq.
BANKERS.?The Bank of England and its Branches.
CONSULTING ACTUARY.-George King, Esq., F.F.A, F.I A. SECRETARY.?Philip Grove, Esq.
Prospectuses and all information may be obtained on application to the Secretary, 38, Old Jewry, London, E.C.

				

